# Impact of booster vaccination on the control of COVID-19 Delta wave in the context of waning immunity: application to France in the winter 2021/22

**DOI:** 10.2807/1560-7917.ES.2022.27.1.2101125

**Published:** 2022-01-06

**Authors:** Paolo Bosetti, Cécile Tran Kiem, Alessio Andronico, Juliette Paireau, Daniel Levy-Bruhl, Lise Alter, Arnaud Fontanet, Simon Cauchemez

**Affiliations:** 1 Mathematical Modelling of Infectious Diseases Unit, Institut Pasteur, Université de Paris, CNRS UMR2000, Paris, France; 2 Collège Doctoral, Sorbonne Université, Paris, France; 3 Santé publique France, French National Public Health Agency, Saint-Maurice, France; 4 Haute Autorité de Santé, Saint-Denis la Plaine, France; 5 Emerging Diseases Epidemiology Unit, Institut Pasteur, Université de Paris, Paris, France; 6 PACRI Unit, Conservatoire National des Arts et Métiers, Paris, France

**Keywords:** SARS-CoV-2, vaccination, France, modelling

## Abstract

Europe has experienced a large COVID-19 wave caused by the Delta variant in winter 2021/22. Using mathematical models applied to Metropolitan France, we find that boosters administered to ≥ 65, ≥ 50 or ≥ 18 year-olds may reduce the hospitalisation peak by 25%, 36% and 43% respectively, with a delay of 5 months between second and third dose. A 10% reduction in transmission rates might further reduce it by 41%, indicating that even small increases in protective behaviours may be critical to mitigate the wave.

Most European countries experienced an important rise in severe acute respiratory syndrome coronavirus 2 (SARS-CoV-2) infections and hospitalisations in the autumn of 2021. In response to this resurgence and to the reported partial decay of immunity, countries have started administering vaccine booster doses, relying on different eligibility criteria. Here, we present modelling analyses assessing different administration strategies for booster doses that informed the recommendations of the French National Immunisation Technical Advisory Group (Haute Autorité de Santé) in the context of Metropolitan France.

## Modelling immunity and the impact of vaccines

We extended a deterministic compartmental model presented in detail by Bosetti et al. [1] (see Supplementary Figure S1 for the model diagram). We account for age-specific mixing patterns [2] and for a lower susceptibility to SARS-CoV-2 infection in children (0–9 and 10–17 years-olds are, respectively, 50% and 25% less susceptible than adults) [3,4]. The model considered the epidemic wave caused by the SARS-CoV-2 Delta variant (Phylogenetic Assignment of Named Global Outbreak (Pango) lineage designation (B.1.617.2) and did not capture the future impact of the Omicron variant (B.1.1.529).

**Figure 1:**
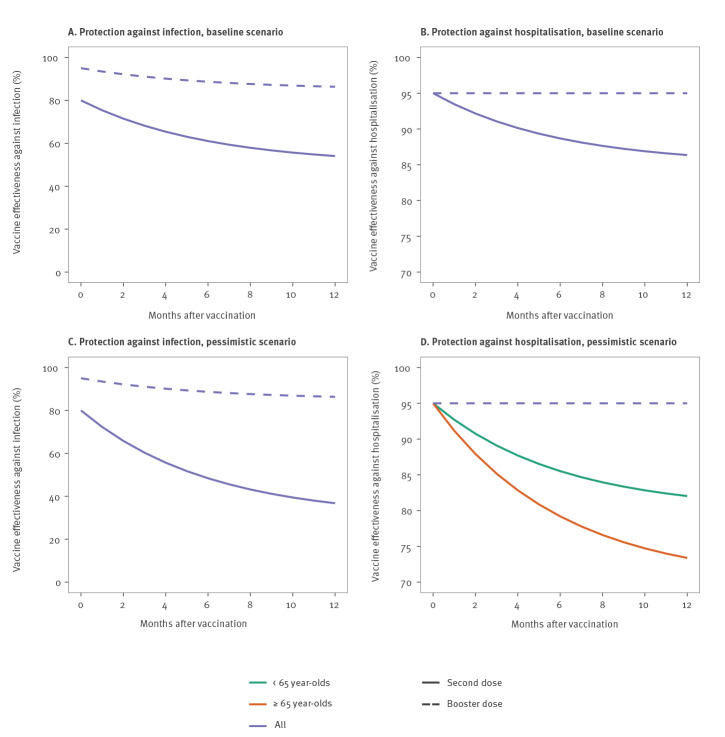
Assumptions regarding SARS-CoV-2 vaccine effectiveness over time

Our model explicitly accounted for the decay of vaccine effectiveness [5] (Figure 1). In our baseline scenario, we assumed that after 6 months on average, vaccine effectiveness against infection decreased from 80% to 50% [5] and vaccine effectiveness against hospitalisation decreased from 95% to 85%. In a more pessimistic scenario, vaccine effectiveness against infection decreased to 30%, whereas protection against hospitalisation decreased to 80% in those younger than 65 years and to 70% in people 65 years and older. Assumptions regarding vaccine effectiveness are detailed in Supplementary Tables S1 and S2.

SARS-CoV-2: severe acute respiratory syndrome coronavirus 2.

Note that the y axes are on different scales.

We assumed that 7 days after receiving a booster dose, effectiveness against infection and hospitalisation is 95%. After 6 months on average, protection against infection drops to 85% (protection against hospitalisation remains constant). We also explored a scenario in which the booster confers 99% protection against hospitalisation. In all scenarios, we assumed that fully vaccinated individuals (with or without a booster dose) and individuals previously infected are half as infectious as individuals with no prior history of infection or vaccination.

We assumed that infected individuals who have not been vaccinated are fully protected against reinfection for 3 months on average. After this, their protection against infection drops to 85% and, after an additional 6 months on average, to 60%, while protection against hospitalisation drops to 90% and 85%, respectively.

## Administration of vaccine doses

We assumed that individuals are eligible for a booster dose 5 months after their second dose if they are aged ≥ 65, ≥ 50 or ≥ 18 years. We also explored scenarios where they are eligible 4 or 6 months after their second dose. Among eligible individuals, we assumed that 80% of ≥ 50 and 50% of 18–49 year-olds accept the booster dose. We also explored a scenario with an acceptance of 95% for all. We assumed that at most 400,000 or 600,000 doses are administered per day. The future roll-out of second doses was captured with an exponential decrease model (see Supplementary Figure S2 for further detail).

Children aged 5–11 years remained unvaccinated in our baseline scenario. In a sensitivity analysis, this age group was vaccinated from 15 December 2021 at a pace of 50,000 first doses per day with an acceptance of 70%, regardless of the booster roll-out pace.

## Epidemiological scenarios during winter

In our baseline scenario, we assumed that the reproduction number R_0_ (mean number of persons infected by a case accounting for the effect of control measures if there was no population immunity) will remain equal to the one we estimated between 2 and 22 November 2021 (R_0_ = 4.8; 95% credible interval (CrI): 4.6–5.0). In sensitivity analyses, we assumed transmission rates decrease by 10% or 20% from 1 December 2021 as the population compliance with protective behaviours increases and the government strengthens non-pharmaceutical measures in response to the epidemic progression. All scenarios accounted for seasonal variations (33% amplitude in R_0_ between summer and winter) [6]. We assumed that the hospitalisation probability for the SARS-CoV-2 Delta variant is 50% higher than for Alpha (B.1.1.7) [7], whereas the Alpha variant is 42% more severe than previously circulating strains [8]. A detailed description of the model and parameters is reported in the Supplement.

## Strategies targeting different age groups

In our baseline scenario, we therefore assumed (i) constant R_0_ from 22 November 2021, (ii) a minimum 5 months delay between the second and third vaccine dose, (iii) a maximum of 400,000 doses administered per day from 1 December 2021 and (iv) a booster acceptance of 80% and 50% among ≥ 50 and 18–49 year-olds, respectively. We then explored in sensitivity analyses how results changed when we modified these assumptions.

**Figure 2:**
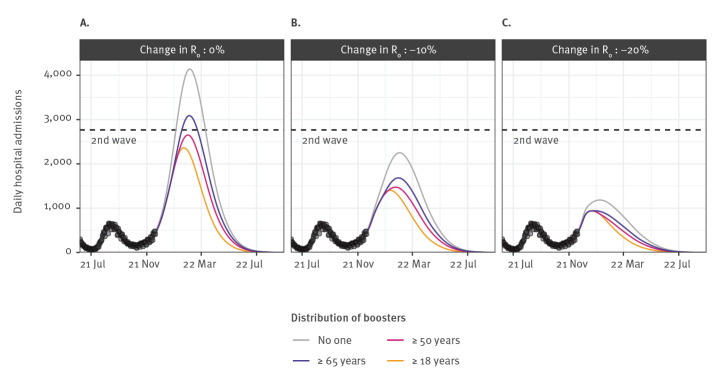
Expected impact of different SARS-CoV-2 vaccine boosting strategies on the daily number of hospital admissions, France, July 2021–September 2022

If no booster doses are distributed to the population, we anticipate a peak of 4,140 daily hospital admissions and a cumulative number of 380,000 hospitalisations between 1 November 2021 and 1 May 2022 in Metropolitan France (Figure 2A). However, if boosters are distributed to those aged ≥ 65, ≥ 50 or ≥ 18 years, the hospitalisation peak is reduced, respectively, by 25%, 36% and 43% and the cumulative number of hospitalisations by 23%, 33% and 44%, respectively (Figure 2A).

SARS-CoV-2: severe acute respiratory syndrome coronavirus 2.

Daily number of hospital admissions assuming transmission rates from 1 December 2021 remain unchanged (panel A), are reduced by 10% (panel B) or are reduced by 20% (panel C). We explored strategies where booster doses are not distributed, distributed in those aged 65 and older only, those aged 50 and older and those aged 18 and older.

## Strengthening protective behaviours

When we considered individuals 18 years and older eligible for a booster, reducing R_0_ by 10% and 20% from 1 December led, respectively, to a reduction in the hospitalisation peak of 41% and 60% and a reduction in the cumulative number of hospitalisations of 34% and 59%, relative to the scenario without reduction in R_0_ (Figures 2B-C).

## Logistical characteristics of the booster vaccination campaign

**Figure 3:**
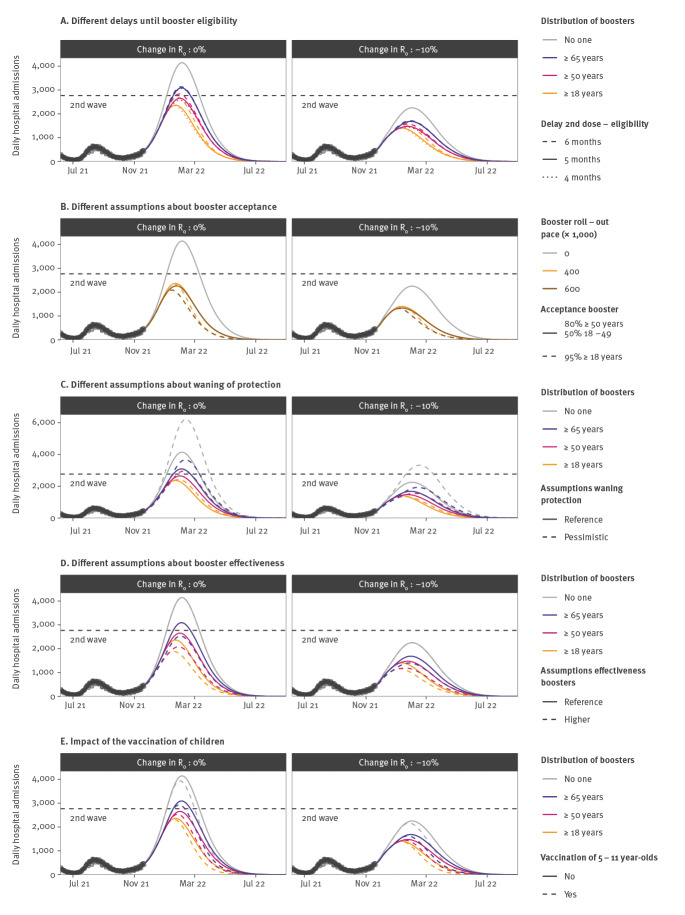
Sensitivity analyses exploring different SARS-CoV-2 booster and vaccine eligibility criteria, booster and vaccine effectiveness and booster acceptances, France, July 2021–September 2022

The reduction in the peak number of hospitalisations increased from 35% for a delay of 6 months between the second and third dose to 43% for a delay of 4 or 5 months (Figures 3A). Further impact could be achieved by increasing the number of doses administered daily along with acceptance of the booster. For a maximum of 600,000 doses administered daily and an acceptance of 95% among those 18 years and older, the reduction of the hospitalisation peak and of the cumulative number of hospitalisations is 50% and 54%, respectively (Figure 3B), compared with 43% and 44%, respectively, in our baseline scenario.

SARS-CoV-2: severe acute respiratory syndrome coronavirus 2.

Panel A: Impact of changing the delay between receiving a second dose and being eligible for a booster. Panel B: Impact of an increased acceptance of booster doses and/or a faster roll-out of boosters. Panel C: Impact of more pessimistic assumptions about the waning of protection conferred by vaccination. Panel D: Impact of a higher effectiveness of booster doses. Panel E: Impact of the vaccination of children. In panel B, the trajectories are presented under the assumption that booster doses are distributed in those 18 years and older.

## Vaccine effectiveness

For more pessimistic assumptions about immunity decay, we expect a higher peak in the absence of boosters, and a larger relative reduction of peak size induced by the booster (61% compared with 43% in the baseline scenario when ≥ 18 year-olds are targeted; Figure 3C). A more effective booster (99% reduction against hospitalisation) would also lead to larger reductions (55% when ≥ 18 year-olds are targeted; Figures 3D).

## Vaccination of children

Vaccinating 5–11 year-old children from mid-December would have limited impact on the hospitalisation peak of the wave in winter 2021/22 (2% reduction compared with a scenario where children are not vaccinated; Figure 3E). It would reduce infections and hospitalisations among 0–9 year-olds by 21% and 22%, respectively, between 1 November 2021 and 1 May 2022. Assumptions regarding the relative infectivity/susceptibility in children (Supplementary Table S3) can influence our estimates but the impact on the overall peak in hospitalisations remains low in all scenarios.

## Discussion

Given the reported immunity decay [5], we found that the fast administration of booster doses to adults 18 years and older vaccinated at least 5 months ago can substantially mitigate the impact of the pandemic wave associated with the SARS-CoV-2 Delta variant in France in winter 2021/22. This result is corroborated by the experience in Israel, where a large pandemic wave could be controlled with such an approach [9].

Administering boosters to all adults has a larger impact than targeting older adults only because of (i) the important decay of protection against infection and (ii) the important contribution of young adults to SARS-CoV-2 spread [10]. In this context, increasing their protection reduces community transmission, indirectly protecting frail individuals. Small reductions in R_0_ due to the strengthening of protective behaviours can have an important effect on epidemic dynamics.

While our results may inform recommendations in other European countries, they are sensitive to country-specific features. Firstly, France has achieved high two-dose vaccine coverage (ca 80% of teenagers and 90% of adults). In countries with lower vaccine coverage, boosting vaccinated individuals should have a more limited impact, since unvaccinated individuals contribute more to disease spread and hospitalisations. Secondly, the French population was mostly vaccinated with the SARS-CoV-2 Comirnaty vaccine (BNT162b2 mRNA, BioNTech–Pfizer, Mainz, Germany/New York, United States). For vaccines characterised by larger immunity decay, boosting may lead to larger gains. Finally, the impact of logistical features (e.g. delay between the second and the third dose, maximum number of doses distributed daily) will depend on the timing of second dose distribution relative to the current wave. For example, under the assumption that the vaccine boost has the same impact on the immune system when administered after 4, 5 and 6 months, we found that reducing the delay between doses can provide substantial gains in France because many French people were vaccinated in Summer 2021 (see Supplementary Figure S3 depicting the metropolitan French population eligible through time). Those gains might be more limited if countries achieved high vaccine coverage at a different time.

We find that vaccinating children from mid-December would have little impact on the current hospitalisation wave. This result reflects the late timing of this vaccination with respect to the wave. The impact of vaccinating children could have been substantial if it had started earlier (Supplementary Figure S4). It is therefore important to anticipate the impact beyond the current Delta wave, particularly with the rise of the Omicron variant [11].

We investigated the impact of boosting on the Delta-driven pandemic wave in winter 2021/22. The emergence of the Omicron variant is a cause for concern [11] and will add to the burden anticipated for the Delta variant. The impact of Omicron will depend on its characteristics (transmissibility, severity, immune escape). In any case, measures available to mitigate the Delta wave (booster doses and strengthening of protective behaviours) will also help delay and mitigate this impact.

## Conclusion

The rapid roll-out of booster doses to the population 18 years and older can reduce the impact of the wave caused by the SARS-CoV-2 Delta variant considerably. Small reductions in transmission rates (e.g. from the adoption of protective behaviours) can substantially reduce the stress on the healthcare system.

## Supplementary Data

710000 Supplement
