# Supplementary material for: Impact of booster vaccination on the control of COVID-19 Delta wave in the context of waning immunity: application to France in the winter 2021/22
Source: Euro Surveill. 2022 Jan 6;27(1):2101125. doi: 10.2807/1560-7917.ES.2022.27.1.2101125 (PMC8739339; doi:10.2807/1560-7917.ES.2022.27.1.2101125)
Supplement: Supplement [file 21-01125_CAUCHEMEZ_Supplement.pdf]

Supplementary material of : “Impact of booster vaccination on the control of COVID-19 in the context of waning immunity: Application to France in the autumn-winter 2021-2022”

This supplementary material is hosted by Eurosurveillance as supporting information alongside the article Impact of booster vaccination on the control of COVID-19 in the context of waning immunity: “Application to France in the autumn-winter 2021-2022”, on behalf of the authors, who remain responsible for the accuracy and appropriateness of the content. The same standards for ethics, copyright, attributions and permissions as for the article apply. Supplements are not edited by Eurosurveillance and the journal is not responsible for the maintenance of any links or email addresses provided therein.

Model description

We have extended a deterministic age-structured model presented in detail by Bosetti et al. (Bosetti et al. 2021) to account for the progressive waning of protection provided by vaccination (2 doses or booster vaccination) as well as the protection acquired following a SARS-CoV-2 infection. The model accounts for the distribution of SARS-CoV-2 vaccines (distribution of SARS-CoV-2 first and second vaccine doses, i.e. a complete scheme) and the distribution of boosters as well as the impact of climate on the reproduction number.

The flow diagram of the model is depicted in Supplementary Figure S1.

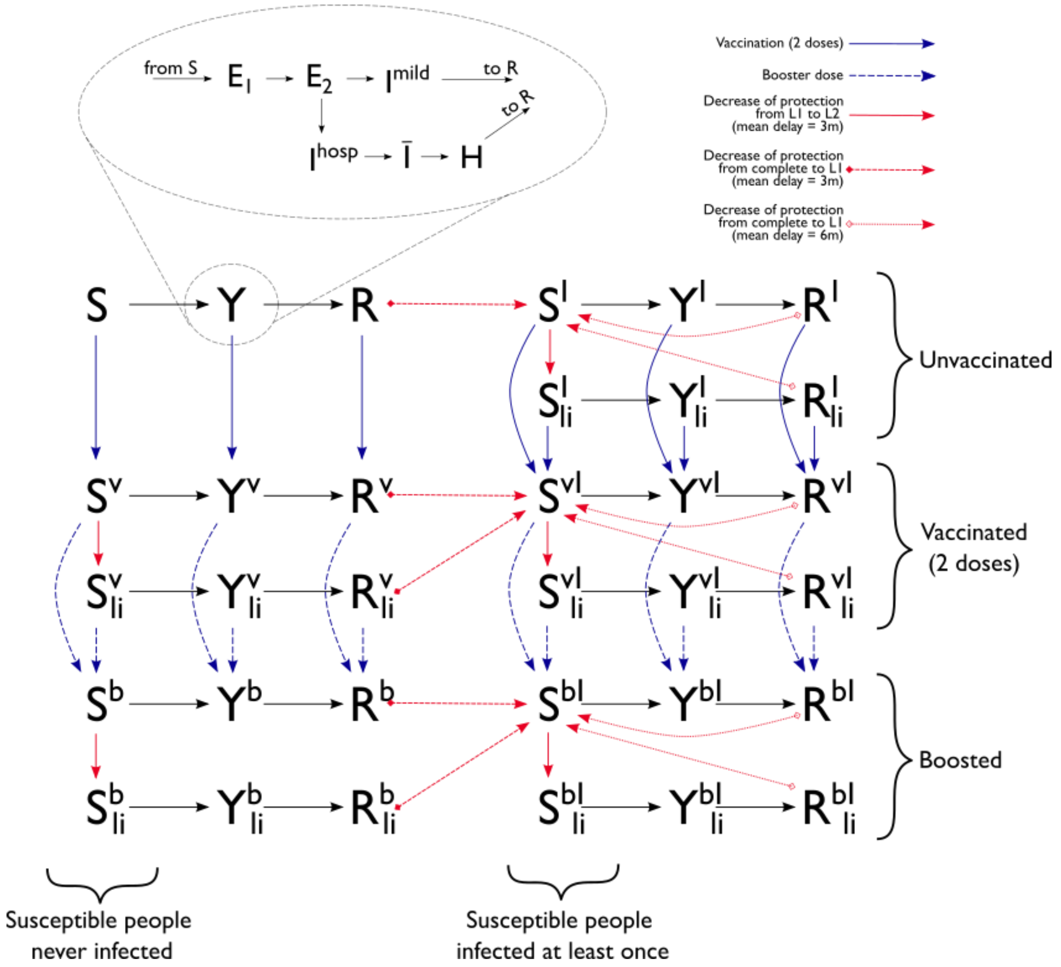

Supplementary Figure S1. Schematic of the model.

Each path denoted by SYR describes the progression of individuals throughout the different stages of the infection (Salje et al. 2020). Susceptible individuals (S) move to the compartment E_1 upon infection. They remain on average 4.0 days in this compartment before moving to the E_2 compartment, in which they become infectious. In E_2, the average length of stay is 1.0 day. They move to the I compartments (I^{mild} for mild infections or I^{hosp} for infections requiring an hospitalization), where they will stay for an average of 3 days. Individuals in the I^{mild} compartment will eventually move to the recovered compartment (R) while individuals in the I^{hosp} compartment move to the $\bar{I}$ compartment before being admitted in hospital (entry in the compartment H). Finally, Individuals in the H compartment will move to the R compartment after an average delay of 13 days. The average length of stay in the R compartment is 3 months following a primary infection, 6 months following a secondary infection. We account for age-specific probabilities of hospitalization as well as the increased severity associated with the Alpha and Delta VOC. We use probabilities of hospitalization estimated in Lapidus et al. (Lapidus et al. 2021) for the strains circulating in 2020 and assume that Alpha is 42% more severe than historical strains (Bager et al. 2021) and Delta is 50% more severe than Alpha (Twohig et al. 2021).

To account for the different immune status in the population, we consider 11 different SYR paths (starting from ${S^a}_b\ {Y^a}_b\ {R^a}_b$). Individuals without a history of prior vaccination or infection are denoted with the path without a subscript (SYR). The superscripts a in (${S^a}_b\ {Y^a}_b\ {R^a}_b$) indicates the history of prior vaccination, boosting or SARS-CoV-2 infection of the different individuals:

- I indicates compartments where individuals were previously infected but never vaccinated.
- v indicates compartments where individuals were vaccinated (2 doses).
- vI indicates compartments where individuals were previously infected and vaccinated (2 doses).
- b indicates compartments where individuals received a booster dose but were never infected.
- bI indicates compartments where individuals were previously infected and received a booster dose.

The subscript *li* indicates compartments where individuals have partially lost the protection acquired following vaccination, boosting, infection or a combination of these. We assume that the waning of protection occurrs on average 6 months after the acquisition of protection.

Individuals who have been vaccinated/boosted or previously infected who are eventually infected follow the same progression throughout the different disease stages as those without a history of prior infections and vaccination. However, we account for a reduced risk of infection upon contact with an infected individual, a reduced risk of being hospitalized as well a lesser infectivity assuming infection compared to unvaccinated and never-infected individuals (see Supplementary Table S1, Supplementary Table S2).

Computing the protection acquired following vaccination through time accounting for waning

Let T be a random variable corresponding to the mean duration before waning of immunity. T follows an exponential distribution with mean $1/\lambda$= 6 months. Let VE_1 and VE_2 respectively denote the levels of vaccine effectiveness before and after waning of vaccine induced protection. The average vaccine effectiveness at time t can be derived as:

$$VE(t) = P[T \leq t] \cdot VE_1 + P[T > t] \cdot VE_2 = (VE_1 - VE_2) \cdot e^{-\lambda t} + VE_2$$

Calibration of the model from February 1st 2020 to June 6th 2021 (historical lineage and alpha VOC periods)

We calibrated our model using the two-step technique outlined in Tran Kiem et al. (Tran Kiem et al. 2021) for the period between February 1st 2020 and June 6th 2021, until the emergence of the Delta VOC in France. To do so, we first fit a one-strain model to daily hospital admissions observed in metropolitan France from February 1st 2020 to January 1st 2021. We then fit a two-strains model to

- daily hospital admissions reported between January 1st, 2021 and June 6th, 2021
- the proportion of cases associated with the Alpha variant obtained from a national survey (Flash) (Santé Publique France 2021)

Assumptions regarding the reduction in the probability of being infected upon contact with an infected individual and the risk of being hospitalized are reported with different immune status in Supplementary Table S1.

Supplementary Table S1: Protection acquired following infection or vaccination between February 1st 2020 to June 6th 2021 (strains circulating during 2020 and Alpha variant period).

	**Protection against infection**		**Protection against hospitalization**	
	**Level of protection L1 (before the decay)**	**Level of protection L2 (after the decay)** (compartments associated with subscript li)	**Level of protection L1 (before the decay)**	**Level of protection L2 (after the decay)** (compartments associated with subscript li)
**Infected*** (Compartments associated with the superscript I)	100%	90%	95%	
**Vaccinated people (2 doses)** (Compartments associated with the superscript v)	80%	60%	95%	
**Infected + Vaccinated people*** (Compartments associated with the superscript vI)	100%	95%	95%	

**After the first infection you are fully protected for 3 months before going to the level of protection L1. After a secondary infection you are fully protected for 6 months before going to the level of protection L1.*

Calibration of the model from June 6th 2021 to November 20th 2021 (emergence of Delta)

To account for the rapid spread of the Delta variant in the metropolitan French population, we fit a two-strains (Alpha and Delta) model to the daily number of hospital admissions and the percentage of Delta VOC among all case observed in metropolitan France (Santé publique France 2021), as in the previous stage. The initialization of this two-strain model is achieved by populating the Alpha and Delta compartment proportionally based on the estimated proportion of Delta variant among infections by june 6th, 2021.

We explore two scenarios regarding the waning of protection acquired following vaccination (an optimistic - baseline - scenario and a pessimistic scenario). Assumptions regarding the

reduction in the probability of being infected upon contact with an infected individual and the risk of being hospitalized are reported with different immune status in Supplementary Table S2 during this time-period.

SupplementaryTable S2: Protection acquired following infection or vaccination from June 6th 2021 (Delta period).

		**Protection against infection**		**protection against hospitalization**	
		**Level of protection L1 (before the decay)**	**Level of protection L2 (after the decay)** (compartments associated with subscript *Ii*)	**Level of protection L1 (before the decay)**	**Level of protection L2 (after the decay)** (compartments associated with subscript *Ii*)
**Infected*** (Compartments associated with the superscript *I*)	**Optimistic and pessimistic**	85%	60%	90%	85%
**Vaccinated people (2 doses)** (Compartments associated with the superscript *v*)	**Optimistic (Baseline)**	80%	50%	95%	85%
	**Pessimistic**	80%	30%	95%	80% for < 65y.o<br>70% for ≥ 65y.o.
**Infected + Vaccinated people*** (Compartments associated with the superscript *vI*)	**Optimistic and pessimistic**	95%	85%	95%	

*After the first infection you are fully protected for 3 months before going to the level of protection L1. After a secondary infection (or more) you are fully protected for 6 months before going to the level of protection L1.

Distribution of first vaccine doses

We calibrate an exponential decrease model on the curve of primo-vaccinations by age between October 15th and November 5th, 2021. We assume that the daily number of primo-vaccinations by age will continue to steadily decline at this rate. Supplementary Figure S2 shows the expected dynamics of the proportion of the people that will receive their first dose of vaccine in the different age groups. By December 31st, 2021, we expect 91% of people over 18 y.o. to be vaccinated, and 79% of those aged 12-17. Among those over 18, the projected proportion of people vaccinated by age is relatively homogeneous, with a maximum of 96% for 75-79 y.o. age group. We calculate the number of second-dose vaccinations based on this evolution by assuming a three-week interval between the first and second dose.

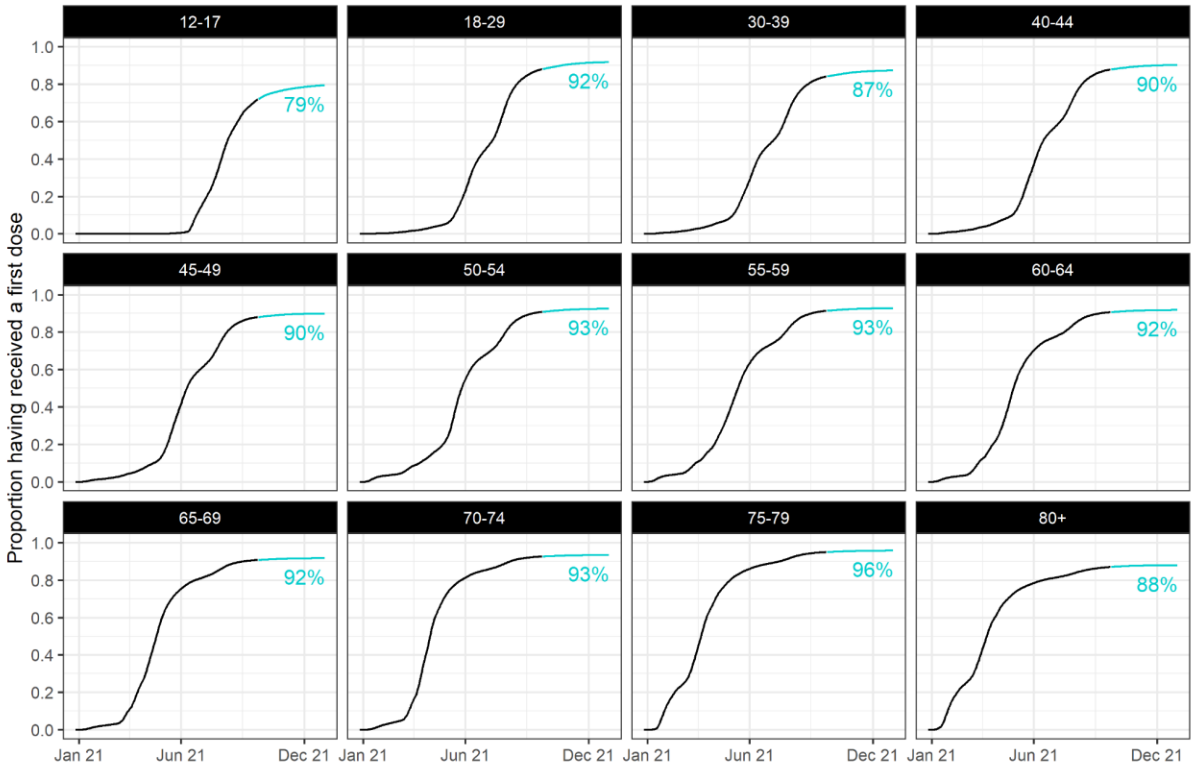

Supplementary Figure S2: Proportion of the French population having received a first dose in the different age groups by December 31st, 2021. The black lines correspond to the vaccination data and the blue one to the projections using our exponential decrease model. The coverages reported in percent correspond to the predicted proportion having received a first dose in the different age groups by December 31st, 2021.

Eligibility to booster doses

Supplementary Figure S3 shows the cumulative number of persons that are eligible for a booster dose, under the assumption of a 5-month delay between the second dose and the booster.

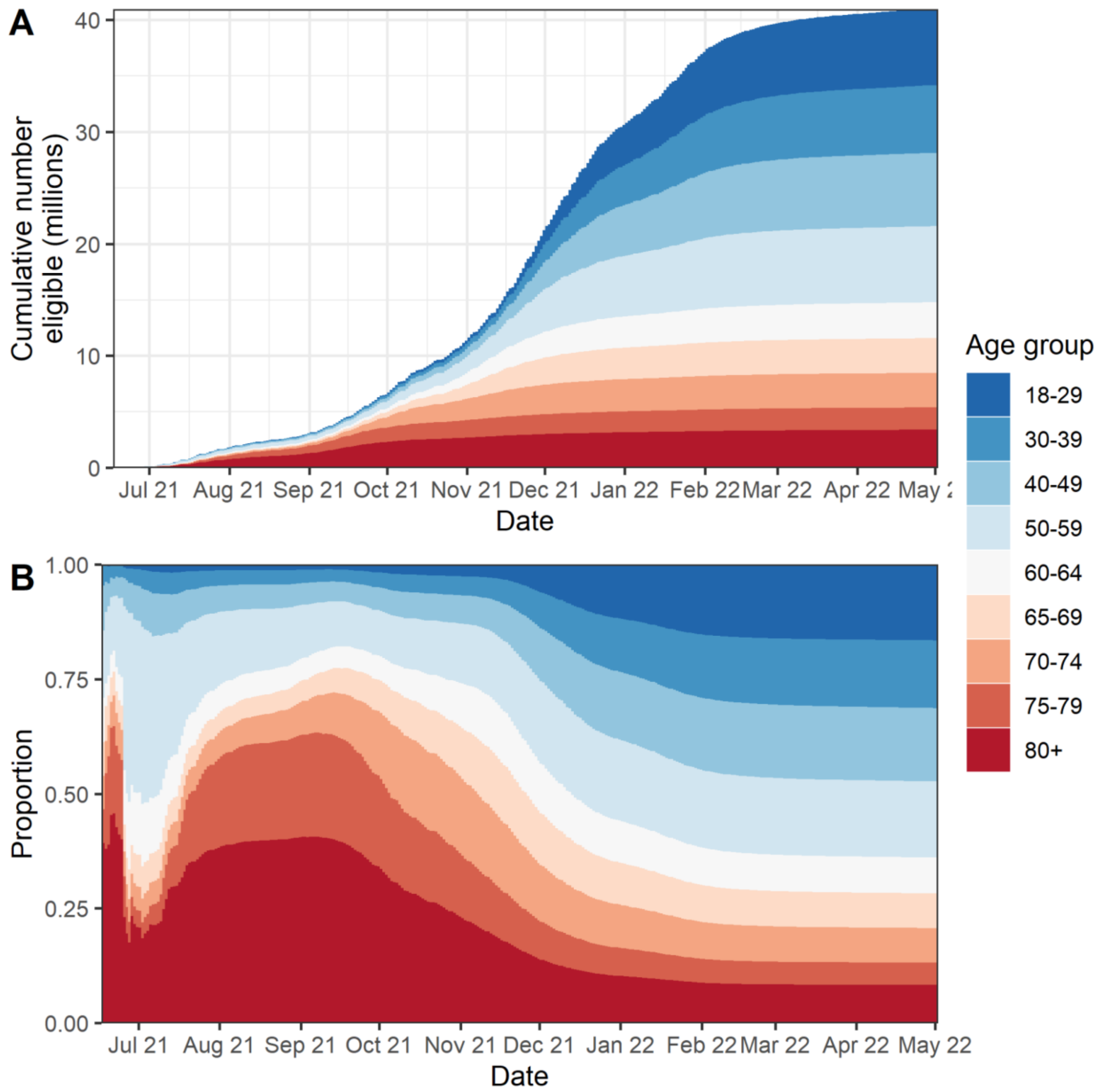

Supplementary Figure S3: Population eligible to a booster dose assuming a 5 month-delay between second doses and eligibility in metropolitan France. (A) Cumulative number eligible to a booster dose in the different age groups. (B) Proportion of eligible individuals by age group through time.

Counterfactual analysis assuming the vaccination of children started on September 1st, 2021

We present in Supplementary Figure S4 the retrospective impact the vaccination of children could have had assuming the roll-out of first doses started in children aged 5-11 y.o. on September 1st, 2021 with a vaccine acceptance of 70% in this group and assuming first doses are being administered at a pace of 50,000 per day. In this scenario, the vaccination of children might have reduced hospitalisation peak by 81% and the number of infections and hospitalisations among 0-9 y.o. children by 83% and 84%, respectively. Table S3 shows how these results would be modified under the assumption that children aged 0-9 y.o. are 50% less infectious than adults.

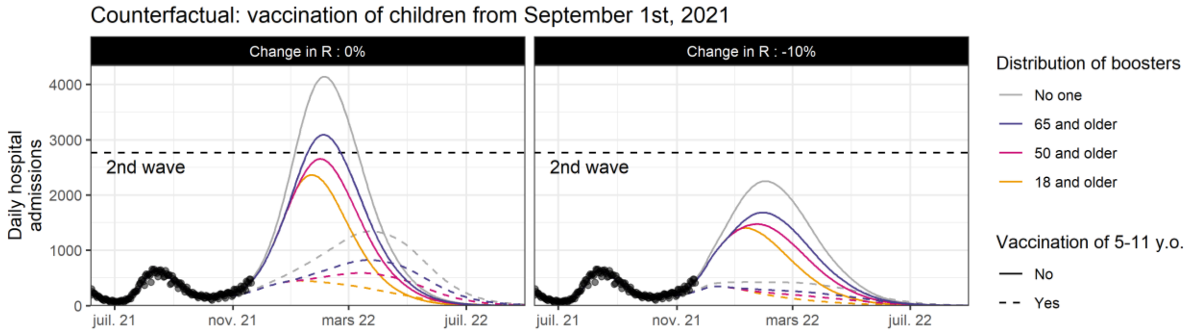

Supplementary Figure S4: Counterfactual analysis of the impact of initiating the vaccination of children aged 5-11 y.o. on September 1st, 2021. Daily hospital admissions for different groups targeted for the roll-out of boosters (colors) and assuming children are vaccinated are not starting from September 1st, 2021 (dashed/plain lines). We explore scenarios where transmission rates remain unchanged after December 1st, 2021 and where they are reduced by 10%.

Supplementary Table S3: Sensitivity analysis for the baseline and counterfactual scenario, assuming children aged 0-9 are as infectious, 50% less infectious than adults or only 25% less susceptible than adults compared to 50% in our baseline scenario.

	**Reduction of the peak in daily hospital admissions**		**Reduction of the cumulative number of infections in children aged 0 - 9 y.o.**		**Reduction of the cumulative number of hospitalizations in children aged 0 - 9 y.o.**	
**Start of the vaccination in 5-11 y.o.**	**Dec 15th (reference)**	**Sep 1st (counterfactual)**	**Dec 15th**	**Sep 1st**	**Dec 15th**	**Sep 1st**
**Children as infectious as adults and 50% less susceptible than adults (reference)**	2%	81%	21%	83%	22%	84%
**Children 50% less infectious than adults and 50% less susceptible than adults**	2%	81%	21%	83%	22%	85%
**Children as infectious as adults and 25% less susceptible than adults**	<1%	75%	7%	66%	8%	70%

References

Bager, Peter, Jan Wohlfahrt, Jannik Fonager, Morten Rasmussen, Mads Albertsen, Thomas Yssing Michaelsen, Camilla Holten Møller, et al. 2021. "Risk of Hospitalisation Associated with Infection with SARS-CoV-2 Lineage B.1.1.7 in Denmark: An Observational Cohort Study." *The Lancet Infectious Diseases* 21 (11): 1507–17.

Bosetti, Paolo, Cécile Tran Kiem, Alessio Andronico, Vittoria Colizza, Yazdan Yazdanpanah, Arnaud Fontanet, Daniel Benamouzig, and Simon Cauchemez. 2021. "Epidemiology and Control of SARS-CoV-2 Epidemics in Partially Vaccinated Populations: A Modeling Study Applied to France," September. https://hal-pasteur.archives-ouvertes.fr/pasteur-03272638/document.

Lapidus, Nathanael, Juliette Paireau, Daniel Levy-Bruhl, Xavier de Lamballerie, Gianluca Severi, Mathilde Touvier, Marie Zins, Simon Cauchemez, Fabrice Carrat, and SAPRIS-SERO study group. 2021. "Do Not Neglect SARS-CoV-2 Hospitalization and Fatality Risks in the Middle-Aged Adult Population." *Infectious Diseases Now* 51 (4): 380–82.

Salje, Henrik, Cécile Tran Kiem, Noémie Lefrancq, Noémie Courtejoie, Paolo Bosetti, Juliette Paireau, Alessio Andronico, et al. 2020. "Estimating the Burden of SARS-CoV-2 in France." *Science* 369 (6500): 208–11.

Santé Publique France. 2021. "Enquêtes Flash : évaluation de La Circulation Des Variants Du SARS-CoV-2 En France." 2021. https://www.santepubliquefrance.fr/etudes-et-enquetes/enquetes-flash-evaluation-de-la-circulation-des-variants-du-sars-cov-2-en-france.

Santé publique France. 2021. "Données de Laboratoires Pour Le Dépistage : Indicateurs Sur Les Mutations." https://www.data.gouv.fr/fr/datasets/donnees-de-laboratoires-pour-le-depistage-indicateurs-sur-les-mutations/.

Tran Kiem, Cécile, Clément R. Massonnaud, Daniel Levy-Bruhl, Chiara Poletto, Vittoria Colizza, Paolo Bosetti, Arnaud Fontanet, et al. 2021. "A Modelling Study Investigating Short and Medium-Term Challenges for COVID-19 Vaccination: From Prioritisation to the Relaxation of Measures." *EClinicalMedicine* 38 (August): 101001.

Twohig, Katherine A., Tommy Nyberg, Asad Zaidi, Simon Thelwall, Mary A. Sinnathamby, Shirin Aliabadi, Shaun R. Seaman, et al. 2021. "Hospital Admission and Emergency Care Attendance Risk for SARS-CoV-2 Delta (B.1.617.2) Compared with Alpha (B.1.1.7) Variants of Concern: A Cohort Study." *The Lancet Infectious Diseases*, August. https://doi.org/10.1016/S1473-3099(21)00475-8.
